# Whole genome sequence of five strains of *Spiroplasma citri* isolated from different host plants and its leafhopper vector

**DOI:** 10.1186/s13104-020-05160-9

**Published:** 2020-07-03

**Authors:** Raymond Yokomi, Rachel Rattner, Fatima Osman, Yogita Maheshwari, Vijayanandraj Selvaraj, Deborah Pagliaccia, Jianchi Chen, Georgios Vidalakis

**Affiliations:** 1 United States Department of Agriculture -Agricultural Research Service, San Joaquin Valley Agricultural Sciences Center, Parlier, CA 93648 USA; 2 Department of Plant Pathology, University of California, Davis, CA 95616 USA; 3 Department of Botany & Plant Sciences, University of California, Riverside, CA 92521 USA; 4 Department of Microbiology and Plant Pathology, University of California, Riverside, CA 92521 USA

**Keywords:** Citrus stubborn disease, Brittle root disease, Leafhopper vectors, Genome sequencing and annotation, Spiroplasmas, *Circulifer*

## Abstract

**Objectives:**

*Spiroplasma citri* is a bacterium with a wide host range and is the causal agent of citrus stubborn and brittle root diseases of citrus and horseradish, respectively. *S. citri* is transmitted in a circulative, persistent manner by the beet leafhopper, *Neoaliturus (Circulifer) tenellus* (Baker), in North America. Five strains of *S. citri* were cultured from citrus, horseradish, and *N. tenellus* from different habitats and times. DNA from cultures were sequenced and genome assembled to expand the database to improve detection assays and better understand its genetics and evolution.

**Data description:**

The whole genome sequence of five strains of *S. citri* are described herein. The *S. citri* chromosome was circularized for all five strains and ranged from 1,576,550 to 1,742,208 bp with a G + C content of 25.4–25.6%. Characterization of extrachromosomal DNAs resulted in identification of one or two plasmids, with a G + C content of 23.3 to 27.6%, from plant hosts; and eight or nine plasmids, with a G + C content of 21.65 to 29.19%, from *N. tenellus*. Total genome size ranged from 1,611,714 to 1,832,173 bp from plants and 1,968,976 to 2,155,613 bp from the leafhopper. All sequence data has been deposited in DDBJ/ENA/GenBank under the accession numbers CP046368-CP046373 and CP047426-CP047446.

## Objective

Spiroplasmas are wall-less, gram-positive bacteria with mobile helical cells and can infect numerous organisms including plants, insects, mites, ticks, crustaceans, and mammals. Economically important spiroplasmas in the U.S.A. are plant pathogens such as *Spiroplasma citri*, causal agent of citrus stubborn disease (CSD) [1], brittle root of horseradish (*Armoracia rusticana*) [2], and *S. kunkelli,* causal agent of corn stunt [3]. In California, CSD is endemic [4] and can be a serious disease of citrus [5, 6]. Its incidence ranges from spotty to abundant due to reasons such as, but not limited to, abundance of the *S. citri* vector *Neoaliturus tenellus* (Baker), also known as *Circulifer tenellus*, the beet leafhopper (BLH); proximity of citrus to annual crops and dichotomous hosts infected with *S. citri* which are hosts of the BLH; and semi-arid, hot climate/habitat [4, 7]. To determine any genomic differences that may occur between *S. citri* populations, the bacterium was isolated and cultured from different times and hosts, and *S. citri* DNA purified and subjected to PacBio sequencing. The whole genome sequence was assembled for five strains to add to the two *S. citri* sequences in the public database [8, 9]. The genome sequences reported here will be used for comparative genomics and to better understand the etiology, relationships and evolution among the spiroplasmas. In addition, this genomic data will be used to improve detection assays for *S. citri* from those previously published [4, 10, 11].

## Data description

*Spiroplasma citri* was isolated and grown in LD8 medium [12], triple cloned, and stored at − 80 °C. *S. citri* strain C189 was established in 1972 from a Navel orange tree (*Citrus sinensis* (L.) Osb.) [1] in Riverside, California by grafting to Madam Vinous sweet orange seedlings and maintained *in planta* at the Citrus Clonal Protection Program, University of California, Riverside, California. *S. citri* strain BR-12 was obtained in 1981 from horseradish in Collinsville, Illinois [2]. *S. citri* strain LB319 was isolated in 2007 from a Spring Navel orange tree in Ducor, California. *S. citri* strain BLH-13 was isolated in 2010 from BLH collected from parsley (*Petroselinum crispum*) in Mettler, California*. S. citri* strain BLH-MB was isolated in 2011 from BLH collected from Russian thistle (*Salsola tragus*) in Parlier, California.

Cultures were re-established for this study and total genomic DNA was extracted by CTAB [13]. Sequencing was performed using PacBio (Menlo Park, CA, USA) RS II platform using single molecule real-time (SMRT) cell v3 with sequencing polymerase (P) and chemistry 4.0 v2©—P6C4 [9]. The library was prepared using PacBio Procedure-Preparing > 30 kb libraries using SMRTbell Express Template Preparation Kit according to manufacturer’s specifications. Adapter screening and quality filtering of raw sequencing data were performed using SMRT Analysis (PacBio) with default settings. The *S. citri* genomes yielded between 40,816 and 122,010 reads encompassing a range of 5.4 Mb to 1.7 Gb. The N50 value was between 17,795 and 20,974 bp.

For each of the five *S. citri* strains, filtered subreads established by PacBio were assembled into contigs using Canu 1.8 [14]. To check for contig circularity, ~ 500 bp segments from each end of a contig were used to BLASTn search the PacBio read data. Appropriate reads connecting both ends were used for enclosure. The chromosome and plasmid status of each contig were further confirmed by BLASTn analyses against the GenBank database. The *S. citri* chromosome was circularized for all five strains and ranged from 1,576,550 to 1,742,208 bp, with an average coverage of 59-fold and an average G + C content of 25.4%. Total genome size ranged from 1,611,714 to 1,832,173 from plants and 1,968,976 to 2,155,613 from the BLH. Extrachromosomal DNAs were characterized, which resulted in identification of one or two plasmids from the plant hosts; and eight or nine plasmids from the BLH. The genome sequence data has been deposited in the NCBI database under Accession numbers CP046368-CP046373 and CP047426-CP047446 (Table 1; Bioproject; Data set 1–5). Annotation of each contig was performed by the NCBI Prokaryotic Genome Annotation Pipeline (PGAP) [15] and predicted 38 RNA genes for all strains and between 1908 and 2556 coding sequences. These data extend the sequence database of *S. citri* and should help to improve detection assays for *S. citri* and provide insight on the evolution of plant pathogenic spiroplasmas.

**Table 1:** Overview of data files/data sets

Label	Name of data file/data set	File types (file extension)	Data repository and identifier (DOI or accession number)
Bioproject [16]	*Spiroplasma citri* genome sequencing and assembly	No file type	PRJNA591027 (https://www.ncbi.nlm.nih.gov/bioproject/PRJNA591027)
Data set 1 [17]	SRX7514565: Whole genome sequencing *of Spiroplasma citri* strain C189	.fastq.gz	NCBI Sequence Read Archive (https://www.ncbi.nlm.nih.gov/sra/SRR10843927)
Data set 2 [18]	SRX7196003: Whole genome sequencing *of Spiroplasma citri* strain LB 319	.fastq.gz	NCBI Sequence Read Archive (https://www.ncbi.nlm.nih.gov/sra/SRR10507068)
Data set 3 [19]	SRX7196004: Whole genome sequencing of *Spiroplasma citri* strain BR12	.fastq.gz	NCBI Sequence Read Archive (https://www.ncbi.nlm.nih.gov/sra/SRR10507067)
Data set 4 [20]	SRX7196005: Whole genome sequencing of *Spiroplasma citri* strain BLH-MB	.fastq.gz	NCBI Sequence Read Archive (https://www.ncbi.nlm.nih.gov/sra/SRR10507066)
Data set 5 [21]	SRX7196006: Whole genome sequencing of *Spiroplasma citri* strain BLH-13	.fastq.gz	NCBI Sequence Read Archive (https://www.ncbi.nlm.nih.gov/sra/SRR10507065)
Data file 1 [22]	ASM1058717v1: *Spiroplasma citri* strain C189 Whole genome assembly data	FASTA	NCBI Genbank (Accession: GCA_010587175.1) (https://www.ncbi.nlm.nih.gov/assembly/GCA_010587175.1)
Data file 2 [23]	ASM1058710v1: *Spiroplasma citri* strain LB 319 Whole genome assembly data	FASTA	NCBI Genbank (Accession: GCA_010587105.1) (https://www.ncbi.nlm.nih.gov/assembly/GCA_010587105.1)
Data file 3 [24]	ASM1058705v1: *Spiroplasma citri* strain BR12 Whole genome assembly data	FASTA	NCBI Genbank (Accession: GCA_010587055.1) (https://www.ncbi.nlm.nih.gov/assembly/GCA_010587055.1)
Data file 4 [25]	ASM1058730v1: *Spiroplasma citri* strain BLH-MB Whole genome assembly data	FASTA	NCBI Genbank (Accession: GCA_010587305.1) (https://www.ncbi.nlm.nih.gov/assembly/GCA_010587305.1)
Data file 5 [26]	ASM1058725v1: *Spiroplasma citri* strain BLH-13 Whole genome assembly data	FASTA	NCBI Genbank (Accession: GCA_010587255.1) (https://www.ncbi.nlm.nih.gov/assembly/GCA_010587255.1)

## Limitations

- Contigs that did not clearly associate with the chromosome were designated as putative plasmids.
- Plasmids that were not circularized were assumed to be linear.

## Data Availability

The data described in this Data note can be freely and openly accessed from the NCBI database https://www.ncbi.nlm.nih.gov/bioproject/PRJNA591027 [16]. All sequence data has been deposited in DDBJ/ENA/GenBank under the accession numbers CP046368-CP046373 and CP047426-CP047446. Please see Table 1 and references for details and links to the data. The versions described in this paper are the first versions. PacBio sequencing reads in this study have been deposited in BioProject PRJNA591027, SRR10843927 for C189; SRR10507068 for LB319; SRR10507067 for BR12; SRR10507065 for BLH13; and SRR10507066 for BLH-MB. *S. citri* cultures for BLH13, BLH-MB, BR12, and LB319 have been deposited in ATCC no. SD-7532-7535, respectively. However, ATCC Biorepository Service Agreement 2019-BRS-00049 maintains confidentiality of information regarding these cultures. Information on these cultures are available upon request from the corresponding author.

## Abbreviations

BLH: Beet leafhopper
bp: Base pair
CA: California
CSD: Citrus stubborn disease
CTAB: Cetyltrimethylammonium bromide
IL: Illinois
NCBI: National Center for Biotechnology Information
SMRT: Single-molecule real-time
